# Editorial: Challenges of pharmacoeconomics in global health arena—Contemporary momentum in the early 2020s

**DOI:** 10.3389/fpubh.2023.1189671

**Published:** 2023-04-13

**Authors:** Mihajlo Jakovljevic, Nick Verhaeghe, Kyriakos Souliotis, Kristijan Krstic

**Affiliations:** ^1^Institute of Advanced Manufacturing Technologies, Peter the Great St. Petersburg Polytechnic University, Saint Petersburg, Russia; ^2^Institute of Comparative Economic Studies, Hosei University, Tokyo, Japan; ^3^Department of Global Health Economics and Policy, University of Kragujevac, Kragujevac, Serbia; ^4^Department of Public Health and Primary Care, Faculty of Medicine and Health Sciences, Interuniversity Centre for Health Economics Research, Ghent University, Ghent, Belgium; ^5^Interuniversity Centre for Health Economics Research, Faculty of Medicine and Pharmacy, Vrije Universiteit Brussel, Brussels, Belgium; ^6^Faculty of Social and Political Sciences, University of Peloponnese, Corinth, Greece; ^7^Department of Physical Medicine and Rehabilitation, Faculty of Medical Sciences, University of Kragujevac, Kragujevac, Serbia

**Keywords:** pharmacoeconomics, reimbursement, drugs, medicines, global, costs, pharmaceutical policy, pharmaceutical expenditure

Pharmaceutical markets worldwide expose substantial stratification in their Composite Annual Growth Rates (CAGR) (1). This is best visible when observing landscape diversity among the traditionally wealthier OECD countries and leading Emerging Markets such as the BRICS (Brazil, Russia, India, China, South Africa) and EM7 (BRIC+ Mexico, Indonesia, Turkey) (Jakoljevic et al., 2020). An abundance of seminal evidence documents that accurate GDP growth rates and dynamics of global demand for medical services and pharmaceuticals will remain primarily driven by the Emerging Markets as we approach the 2030s. Long-term investment strategies of the leading brand-name Big-Pharma multinationals are targeted precisely toward these same rapidly developing world regions (2). The epicenter of global economic growth appears to remain in the Western Pacific Asia region up to the middle of the century. The pace of globalization has significantly accelerated since the end of the Cold War Era in 1991 (3). These changes profoundly affected healthcare systems worldwide. Health policymakers increasingly started facing new harsh challenges in their uneasy task to provide universal health coverage and decent equity of access to health care services (4). The most prominent demand-side issues are extended longevity, population aging, the rise of non-communicable diseases and growing patient expectations. Supply-side causes are:

- gains in societal welfare and living standards,
- technological innovation in medicine,
- rapid urbanization in developing world regions.

Successful insurance-based risk-sharing agreements made pharmaceuticals dispensing and health care services provision affordable or virtually free at the point of consumption in most OECD and many middle-income countries (5, 6). The massive build-up of workforce capacities and strengthening of primary care and hospital networks contributed to the “supplier-induced demand” phenomenon (7).

There is direct historical evidence of long-term growth in pharmaceutical and overall health spending in absolute and GDP % terms worldwide (8, 9). The accumulated constraints of outsourcing from skyrocketing costs of care were felt in many areas of clinical medicine, even among the wealthiest societies (10). Cardinal examples of expensive and unaffordable therapeutic areas are orphan drugs indicated to treat rare diseases and targeted biologicals used in autoimmune disorders and cancer (Jakovljevic et al., 2020). Last but not least, many nations experience challenges with access to even essential generic pharmaceuticals (11). This is particularly true among the world's poor and underserved citizens residing in rural and suburban areas of low- and middle-income countries. To a large extent, these difficulties are exacerbated by a lack of evidence-based resource allocation and sustainable financing strategies (12).

For example, hypertension has risen to become the world's second leading cause of death. However, rural China's fragmented three-level medical and healthcare system must provide continuous, coordinated, comprehensive care. As a result of the lack of total health care for hypertensive patients, rural China has a low rate of hypertension control. The purpose of the study conducted by Ke et al. was to investigate the costs and benefits of an integrated care model for hypertension management in rural China using three intervention modes: multidisciplinary teams (MDT), multi-institutional pathways (MIP), and global system budget and performance-based payments (SGB-P4P). The integrated care model with performance-based prepaid payments was the most beneficial intervention in the healthcare delivery system for managing chronic diseases in China. In contrast, the general integrated care model (MDT + MIP) was not cost-effective. The integrated care model (MDT + MIP + SGB-P4P) was proposed for use in community hypertension management in rural China as a continuous, patient-centered care system to improve hypertension management efficiency (13). As a result, it is recommended that an integrated care delivery model could be implemented in the community management of hypertension in rural China, forming “patient-centered” continuous services to improve healthcare system efficiency (Ke et al.).

Researchers from the Indian University, Jaipuria Institute of Management, worked on an intriguing topic involving the pharmaceutical industry's role in building resilient health systems. This study investigated the correlation between the pharmaceutical industry's current sustainability agenda, which is based on the United Nations Sustainable Development Goals (SDGs), the elements of the Joint External Evaluation (JEE) tool, and the three components of the One Health approach. This study provides insight into this area and can assist various government and non-government stakeholders in considering the integration of the pharmaceutical sector to improve health security (Saxena et al.).

According to Ye et al., nivolumab plus ipilimumab demonstrated good clinical benefits compared to chemotherapy in treating unresectable malignant pleural mesothelioma (MPM). The study, conducted by Ye et al., aimed to compare the cost-effectiveness of Nivolumab plus ipilimumab vs. platinum plus chemotherapy for American patients' first-line treatment of MPM. The incremental healthcare costs and QALYs for Nivolumab plus Ipilimumab vs. chemotherapy were $196,604.22 and 0.53, respectively, for a total incremental cost-effectiveness ratio (ICER) of $372,414.28/QALY. The ICER for Nivolumab plus ipilimumab is higher than the theoretical willingness-to-pay threshold of $207,659/QALY in the United States, implying that first-line nivolumab plus ipilimumab for unresectable MPM may not be cost-effective compared to platinum plus chemotherapy (Ye et al.).

Another similar research was conducted. Camrelizumab, the first domestic programmed cell death 1 (PD-1) antibody approved for lung cancer in China, has demonstrated efficacy in patients with non-small-cell lung cancer (NSCLC). Camrelizumab combination therapy was more expensive and provided 0.11 QALYs more than chemotherapy in the base case analysis, 0.12 QALYs more in the subgroup analysis, and 0.34 QALYs more in the scenario analysis. In the base case, the ICER was $63,080 per QALY, $46,311 per QALY, and $30,591 per QALY in the subgroup and scenario analyses, respectively. The results showed that camrelizumab combination therapy is not as cost-effective as first-line therapy for NSCLC patients in China. Camrelizumab combination therapy, on the other hand, has a 62.8% chance of dropping below the WTP threshold and being cost-effective for unselected patients (Xiang et al.).

An intriguing study by Xu et al., using evolutionary game analysis, investigates competition and cooperation among urban and rural medical institutions. The findings indicate that the cooperation mechanism between urban and rural medical institutions is essential for rural medical institution efficiency, government supervision, reward and punishment mechanisms. The study advocates using the internal power of medical institutions to promote collaboration between urban and rural medical and health institutions (Xu et al.).

A budget impact analysis (BIA) is an economic assessment that estimates the financial implications of implementing a new intervention. BIA supplements cost-effectiveness analyses in making informed reimbursement decisions (CEAs) (14). Luo et al. presented 18 BIA studies for anti-diabetic drugs for diabetes mellitus conducted in various countries and regions, including Europe, the United States, Asia, and South America, in their systematic review study. With the emergence of different anti-diabetic drugs, BIA is essential for determining the affordability of implementing a new anti-diabetic drug in a specific health setting.

This Research Topic was created to tackle the core challenges of medicines provision and medical care financing across the Globe (15). Its target is to reveal hidden underlying causes of uneven drug access and the growing proportion of out-of-pocket health spending in many regions (16). In its basis, the Topic belongs to the interdisciplinary sciences of pharmacoeconomics and health economics. Various pharmacoeconomic evaluations and health economics studies are within the Topic scope. Health policy considerations have focused on financing mechanisms, medicines, and affordability of health care aimed at universal access to health care (17).

The editors hope that these significant and diverse subjects will contribute to expanding existing knowledge. Furthermore, they offer an excellent opportunity to discuss the fundamental challenges of pharmaceutical care provision and healthcare financing worldwide (18). A diverse group of authors from academia, the pharmaceutical, medical device and economics industries collaborated to present a comprehensive review of advanced health economics challenges and their peculiarities.

## Author contributions

MJ has prepared the manuscript draft. MJ, NV, KS, and KK have revised it for important intellectual content. All contributed to the article and approved the submitted version.
